# Critical care nutrition and the legacy of Rinaldo Bellomo

**DOI:** 10.1016/j.ccrj.2025.100147

**Published:** 2025-10-14

**Authors:** Adam M. Deane, Sandra L. Peake, Andrew R. Davies, Yasmine Ali Abdelhamid, Moritoki Egi, Marianne J. Chapman, Emma J. Ridley

**Affiliations:** a Department of Critical Care, The University of Melbourne, Australia; b Intensive Care Unit, Royal Melbourne Hospital, Australia; c Department of Intensive Care Medicine, The Queen Elizabeth Hospital, Australia; d Discipline of Acute Care Medicine, The University of Adelaide, Australia; e Australian and New Zealand Intensive Care Research Centre, Monash University, Australia; f Intensive Care Unit, Frankston Hospital, Australia; g Department of Anesthesia and Intensive Care, Kyoto University Hospital, Japan; h Dietetics and Nutrition, Alfred Hospital, Melbourne, VIC, Australia

**Keywords:** Constipation, Critical illness, Energy intake, Enteral nutrition, Glycaemic control, Nutrition therapy

## Abstract

We provide an overview of the monumental impact that Professor Rinaldo Bellomo made in the field of critical illness nutrition and metabolism. We reflect on research he conducted into energy delivery, refeeding hypophosphataemia, carbohydrate metabolism and glycaemia, protein delivery and impact of renal perfusion and nitrogen metabolism, and bowel management during nutritional therapy.

## 1 Introduction

Our friend, mentor, and collaborator Professor Rinaldo Bellomo made a seminal contribution to critical care nutrition. Rinaldo always encouraged challenging the “status quo.” He systematically identified aspects of practice and treatments that were provided to the greatest number of patients; nutrition was one of those interventions. His contribution to nutrition alone has resulted in more than 50 scientific articles, >1800 citations and >18 citations in international policy or guideline documents. While bibliometrics related to Rinaldo's outputs are monumental, these summary quantitative data are insufficient to understand the impact he had on us as a mentor and colleague. Not only was he an endless source of research questions to be studied, but if you had your own inspiration and a novel idea, he would make time to talk through the rationale and possible approaches to studying the issue. His intellect, enthusiasm, generosity and work ethic ensured that many concepts or ideas were nurtured, developed and supported so that the idea became a research question, which became a protocol, a completed study and, eventually, a manuscript. Without him there would be many ideas that remained just that. Within this review, we acknowledge his achievements and mentorship and pay respect to his contributions to nutrition specifically.

### 1.1 Energy delivery

Rinaldo's work led to major advances in our understanding of energy delivery to critically ill patients. In the early years, Rinaldo was bemused at the extent of poor feeding received by critically ill patients, largely due to therapies which have now evolved, such as sedation, ventilation, and fluid management.^1^ Rinaldo mentored numerous healthcare workers to conduct research, including the trainee ICU doctor Andrew Davies, who led a single-centre randomised clinical trial comparing feeding via a jejunal tube placed endoscopically or a standard gastric tube.^2^ The rationale for this trial was, as stated by Rinaldo, “even prisoners receive more nutrition than ICU patients”, with the use of jejunal feeding a potential solution to improve delivery. The results from the single-centre trial indicated that enteral nutrition via a jejunal feeding tube reduced gastric residual volumes and incidence of gastrointestinal intolerance. Rinaldo continued to mentor Andrew, who was then a young intensivist, to lead his first multicentre randomised clinical trial that was endorsed by the Australian and New Zealand Intensive Care Society Clinical Trials Group (ANZICS CTG).^3^ ENTERIC was a major trial in the field comparing early jejunal feeding, with a proprietary self-propelling feeding tube, to standard gastric feeding in critically ill patients. The investigators observed no statistical difference in the proportion of target energy delivered via enteral nutrition and similar clinical outcomes, except for minor gastrointestinal bleeding, which was more frequent in the jejunal feeding group and related to the self-propelling nature of the tube. This trial challenged existing dogma that routine placement of a jejunal feeding tube was beneficial.

As time evolved, Rinaldo wanted to challenge the existing belief that critically ill patients should have energy delivered to approximate the amount of energy that they were predicted to expend. He impishly described clinician's desire to deliver approximately 25 kilocalories per kilogram per day as “gluttony.”^4^ Proponents of this dogma suggested that delivering 100% of expected energy expenditure would increase the likelihood of surviving and improve the quality of survivorship.^5^ Rinaldo championed his colleagues to test whether aiming for 100% of target calories, via an energy dense liquid nutrient formula, was superior to aiming for 70% of this target using routine enteral feed formula.^6^ The TARGET Calories trial remains the largest critical care nutrition trial to blind clinicians, investigators, and patients to group allocation. Due to the pragmatic design and collaborative investigators, which were heavily influenced by Rinaldo, 3957 patients were enrolled within an 18-month period. TARGET Calories established that aiming to deliver 25–30 kilocalories per kilogram per day did not improve any patient-centred outcomes but was associated with increased adverse effects, including gastrointestinal tolerance.^7^^,^^8^ The signal of no benefit with more calories was consistent across all subgroups of patients, regardless of ethnicity, or precipitating illness/injury.^9^^,^^10^ This trial supported recent changes to major international guidelines (2016 American Society for Parenteral and Enteral Nutrition, ASPEN guidelines) which recommendation that, at least in those not considered at “nutritional risk”, providing close to 100% of estimated energy requirements was not required.^11^ Recommendations for a more conservative approach were strengthened soon after publication of TARGET Calories by a separate international guideline group (2019 European Society for Parenteral and Enteral Nutrition, ESPEN guidelines).^12^ It also led to two new research programs with $9.4M funding, featured in NZ media with a readership of 500,000, and has been cited in 46 countries.

### 1.2 Refeeding hypophosphataemia

Rinaldo also advanced our knowledge of management of patients who developed hypophosphataemia when receiving enteral nutrition. In a multicentre open-label clinical trial, Rinaldo and colleagues randomised 339 patients who had a serum phosphate concentration less than 0.65 mmol/L within 72 h of commencing nutrition support to calorie restriction (energy intake to 20 kcal/h for at least 2 days and gradual increase thereafter) or usual care (continuing to try and deliver target rates).^13^ The primary outcome was the number of days alive after ICU discharge censored at day 90. While there was no statistically significant difference in the primary outcome, the between-group difference was 4-9 days more with calorie restriction (95% CI, -2.3 to 13.6), many clinicians worldwide now restrict energy delivery when this occurs.^14^ In addition to calorie restriction, Rinaldo evaluated the role of vitamin B1, or thiamine, for patients with refeeding hypophosphatemia.^15^^,^^16^ In a multicentre open-label clinical trial, Rinaldo and colleagues randomised 90 patients with a serum phosphate concentration less than or equal to 0.65 mmol/L to intravenous thiamine (200 mg twice daily) or usual care.^17^ While clinical outcomes were similar in both groups, Rinaldo established that enteral nutrition using existing commercially available formulae can prevent hypovitamin B1 in the critically ill, and intravenous administration of one dose of 200 mg of thiamine achieves supraphysiological vitamin B1 concentrations.^18^

### 1.3 Carbohydrate metabolism and glucose control

Enteral nutrition, particularly when given via carbohydrate, increases blood glucose concentrations, and Rinaldo generated pivotal insights that have fundamentally reshaped critical care practice. A landmark trial was published in 2001 that reported “intensive insulin therapy” to maintain blood glucose at or less than 6.1 mmol/L in ICU reduced mortality.^19^ Rinaldo was among a group of leading clinician-researchers who highlighted concerns regarding the generalisability of the observations from this single-centre trial.^20^ With Simon Finfer et al., the ANZICS CTG completed the NICE-SUGAR trial.^21^ This trial established that intensive insulin therapy aiming for blood glucose concentrations between 4.4 and 6.1 mmol/L increased mortality when compared to targeting blood glucose concentrations of 6–10 mmol/L. NICE-SUGAR has saved the lives of millions of critically ill patients across the world.^22^

Rinaldo was also among the first to challenge the prevailing orthodoxy that blood glucose targets in critically ill patients should be uniform^23^ He considered it a form of madness that we treat patients irrespective of pre-existing glycaemia and/or diabetes. In a pivotal retrospective observational study, he identified that blood glucose concentrations considered safe, and even beneficial, for critically ill patients without pre-existing diabetes, could be harmful in those with diabetes and chronic hyperglycaemia.^24^ He subsequently observed this signal in a prospective cohort study.^25^ It appeared that while hyperglycaemia was less harmful in those with pre-existing diabetes with chronic hyperglycaemia, hypoglycaemia–both absolute and relative–and glycaemic variability remained strongly associated with poor outcomes in this cohort.[26], [27], [28], [29], [30], [31] Under his leadership a series of pilot studies were conducted^6^^,^[32], [33], [34] which led to the evaluation of a more liberal approach to glucose control in patients with pre-existing diabetes. As an antidote to the madness of treating all patients the same, he suggested the trial acronym of LUCID–because the results would provide clarity after a prolonged period of confusion. This was a multicentre parallel-group open-label clinical trial that randomised 419 patients with type 2 diabetes to intravenous insulin titrated to a target blood glucose range of 10–14 mmol/L or insulin titrated to a target range of 6–10 mmol/L.^35^ The primary outcome was incident hypoglycaemia (<4.0 mmol/L). While the investigators reported that the intervention reduced the frequency of hypoglycaemic events, they did not observe benefit for any patient-centred outcome.^36^

### 1.4 Protein administration, renal perfusion, and nitrogen metabolism

Rinaldo made seminal contributions in our understanding of the effects of protein on renal perfusion and function.

Through his work, we developed a greater understanding of the role of intravenous administration of amino acids. In a multicentre parallel-group phase II trial, Rinaldo et al. randomised 474 patients to receive up to 100 g of intravenous amino acids or usual care (no intravenous amino acids). While amino acid therapy significantly increased estimated glomerular filtration rate and urine output, it did not reduce the duration of renal dysfunction or use of renal replacement therapy.^37^ Whilst this trial did not support the further evaluation of this intervention in patients with acute illness admitted to the intensive care unit, Rinaldo turned to the use of amino acids in perioperative medicine. He conducted a single-centre pilot randomised trial of 69 patients having cardiac surgery, which suggested that this was an intervention worth pursuing.^38^ This encouraged a multinational blinded trial conducted by Rinaldo et al. of intravenous amino acid or placebo in 3511 patients having cardiac surgery.^39^ The intervention reduced the rate of stage I acute kidney injury, with no effect observed on the frequency of renal replacement therapy or longer-term renal function. The proposed mechanisms underlying the physiological effect include recruitment of renal functional reserve and increases in renal blood flow and oxygenation of renal cortex and medulla.^40^^,^^41^ These effects appear to be more prominent in those with existing chronic kidney disease.^42^ These findings established that short-term administration of pharmacological doses of intravenous amino acids in patients having cardiac surgery can reduce renal injury as determined by serum creatinine. It is plausible that an improvement in a kidney biomarker will reduce the frequency of renal failure and the need for renal replacement therapies–although this remains unproven currently.

Rinaldo was a crucial mentor and collaborator in the evaluation of enteral protein provision as nutrition therapy.^43^ He helped establish that local practice was to deliver less protein than recommended in the international clinical practice guidelines.^44^ As was done in TARGET Calories, he promoted a methodical approach, and initially established that it was also feasible to compare usual care protein delivery to the augmented protein recommended in guidelines.^45^ His guidance, encouragement, and mentorship were pivotal to the conduct of the TARGET Protein trial.[46], [47], [48] This trial used a cluster-randomized, crossover, open-label design to compare the use of augmented protein (100 g protein/L) to usual protein (63 g protein/L) formula. A total of 3397 patients were included from 8 ICUs over a 12-month period and augmenting enteral protein delivery did not increase days free of the index hospital and alive at day 90 (mean difference = -1.97 (95% CI, -7.24 to 3.30) days.^48^ These results challenged existing expert convictions; and provide clinicians with considerable confidence that augmenting enteral protein to >1 g/kg body weight/day is of no benefit and may even be harmful.

Rinaldo was particularly interested in the physiological effects of dietary protein and how best to feed patients with renal dysfunction.^49^^,^^50^ He observed that there was marked amino acid loss and negative nitrogen balance in patients on renal replacement therapy,^51^ and augmenting enteral protein could reduce the negative nitrogen balance that occurred during critical illness. However, this increased serum urea concentrations and the amount of haemofiltration required to control uraemia^52^

### 1.5 Bowel management

Rinaldo wanted to improve our understanding about a consequence of enteral nutrition, that being bowel actions (or lack thereof) in critically ill patients. He was bewildered with how frequently patient's bowel motions were discussed on the ward round, as he considered nondefaecation the physiological response to critical illness.^53^ Indeed, he thought the fixation of healthcare workers on non-defaecation, which he felt was erroneously referred to as constipation, was irrational.^54^ He wanted to challenge the dogma that critically ill patients needed to have laxative prophylaxis alongside enteral nutrition, as he observed this caused diarrhoea and complications, such as insertion of faecal management devices, that were unpleasant and likely unnecessary for patients.^55^^,^^56^

## 2 Current gaps in our knowledge and his legacy to continue

Rinaldo challenged us to better understand how nutrition should be provided during critical illness. He did this by asking us to question our own practice and assumptions; and he used humour and history lessons to engage us. Tropical Cyclone Alfred caused the 2024 ANZICS CTG Noosa meeting to be prematurely abandoned. Tragically, and unbeknownst to anyone, this would be Rinaldo's final Noosa meeting. Fortuitously, Rinaldo was programmed in the first session of the first day, and gave what was his final research pitch to a capacity audience. Rinaldo proposed “Management & Evaluation of Gluttony Acceptance during Feeding Enterally with Excessive Dose,” with the playful abbreviation of “MEGA-FEED”. This was followed by a similar proposal regarding laxative use and defaecation, with the prudent abbreviation of “MEGA-LAX”. In only a way Rinaldo could, he put to the audience that gluttony and excess (calories, protein, and laxatives) were pivotal questions that must be tackled; and we must find out whether restricting calories, protein, and laxatives/stool softeners during critical illness improves patient outcomes. He also wanted us to understand whether certain nutrition therapies could attenuate muscle loss. He challenged us to study whether specific amino acid/s, rather than mixed protein, could reduce muscle loss. This led to hours being spent trying to devise a study title that could be abbreviated to Kratos, who is the divine personification of strength according to Greek mythology! He also wanted us to determine whether the use of exogenous mixed amino acids as a short-term pharmacological treatment, rather than longer term nutrition therapy, improves outcomes that are important to patients. He urged us to understand whether nondefaecation was a physiological response to critical illness, and whether the practice of prescribing prophylactic bowel regimens when commencing nutrition therapy is an approach best avoided. Finally, he also wanted us to understand whether, after the acute phase of illness resolves, could more nutrition accelerate recovery?

## 3 Summary

We have been incredibly fortunate to know Rinaldo Bellomo as a friend, mentor, and collaborator. His contribution to the entire field of medicine, which included critical care nutrition, was immense. We will never see his like again.

## Conflicts of interest

The authors declare that they have no known competing financial interests or personal relationships that could have appeared to influence the work reported in this paper.
